# Role of Femoral Artery Access Characteristics and Female Sex in In-Hospital Complications for Patients Undergoing Recanalization of Chronic Total Occlusions

**DOI:** 10.3390/jcm14134496

**Published:** 2025-06-25

**Authors:** Kevin Hamzaraj, Caglayan Demirel, Antonia Domanig, Senta Graf, Mariann Gyöngyösi, Christian Hengstenberg, Bernhard Frey, Rayyan Hemetsberger

**Affiliations:** Department of Internal Medicine II, Division of Cardiology, Medical University of Vienna, 1090 Vienna, Austria

**Keywords:** cardiovascular disease, percutaneous coronary intervention, chronic total occlusion, complications, gender

## Abstract

**Background:** Percutaneous coronary intervention (PCI) for chronic total occlusion (CTO) remains a complex procedure that requires advanced operator skills and dedicated devices. Despite increased success rates in experienced centers, the in-hospital complications of CTO PCI remain notably high. Female patients undergoing CTO PCI are reported to experience higher rates of complications; however, the underlying mechanisms remain inadequately defined. **Methods:** We prospectively enrolled consecutive patients undergoing CTO PCI at our university-affiliated tertiary care center over 4 years (2018–2021), aiming to elucidate sex-based disparities in in-hospital complications. In addition, we investigated the impact of angiographic femoral artery metrics on in-hospital complications. **Results:** Among 271 patients who underwent antegrade or retrograde CTO PCI, 222 (81.9%) were men and 49 (18.9%) women. Female patients were significantly older (67 ± 11 vs. 72 ± 12 years; *p* = 0.005) and had a comparable lesion complexity. Women exhibited smaller femoral artery diameters, more side branches at the puncture area and higher bifurcations. In-hospital complications occurred more frequently in women compared to men (16.3% vs. 6.8%; *p* = 0.044). Female sex independently predicted in-hospital complications (OR = 2.92; CI 1.07 to 7.60; *p* = 0.024), yet lost significance after adjustment for femoral artery characteristics. Maximal femoral artery diameter (OR = 0.30, 95% CI: 0.17 to 0.50, *p* < 0.001) and side-branch density (OR = 2.45, 95% CI: 1.26 to 5.20, *p* = 0.012) independently predicted in-hospital complications. **Conclusions:** Female patients undergoing CTO PCI are at increased risk for procedural complications, likely driven by femoral artery anatomical differences. Detailed pre-procedural assessment of femoral artery metrics may improve patient selection, procedural planning, and outcomes, particularly among women.

## 1. Introduction

Percutaneous coronary intervention (PCI) for complex coronary artery disease has witnessed technical advancements in the last decades, yet chronic total occlusions (CTOs) remain among the most technically demanding subsets of coronary lesions. Chronic total occlusions (CTOs) are defined as complete coronary occlusions with a Thrombolysis in Myocardial Infarction (TIMI) score of 0 persisting for at least three months. They present substantial challenges due to low success rates and high complication rates among coronary interventions and require the use of dedicated techniques, operator skills and devices [1,2,3]. Advances in device technology and revascularization strategies have led to increased success rates in contemporary CTO PCI—with experienced centers achieving up to 90% technical success rates [4,5,6,7]. Nevertheless, the incidence of periprocedural complications related to CTO PCI remains substantial, underscoring the need for predictors to guide patient selection and risk stratification [4,8,9].

Age and sex have been extensively investigated as predictors for short- and long-term outcomes across various PCI populations, including all-comer cohorts, acute coronary syndrome, left main coronary artery and CTO [10,11,12,13,14,15]. Some scoring systems integrate female sex as a predictor for long-term mortality and adverse events following PCI [16,17]. However, the impact of sex on contemporary CTO PCI remains poorly understood and the underlying mechanisms remain unclear [10,12,14,18,19]. Women tend to have smaller vessels and lower body mass index (BMI), suggesting anatomical disparities might significantly contribute to their elevated procedural risks and should be carefully considered in CTO PCI [19,20]. However, the role of femoral access site characteristics has been less frequently studied in this context given its potential association to procedural outcomes and complications [21,22,23].

In the present study, we sought to assess the sex differences in in-hospital complications following contemporary CTO PCI and aimed to identify causal relationships within clinical characteristics. In addition, we evaluated for the first time the impact of femoral artery access site characteristics on in-hospital complications in patients undergoing CTO PCI.

## 2. Methods

### 2.1. Study Population and Interventions

We analyzed 271 consecutive CTO PCI procedures between January 2018 and August 2021 as part of a prospective single-center observational registry. Ethical approval was granted by the local institutional review board (Ethikkommission der Medizinischen Universität Wien, EK-Number 1448/2018), and the study adhered to the Declaration of Helsinki. PCI procedures were performed following the hybrid algorithm approach, guided by lesion complexity and operator discretion [24]. CTO was defined as angiographically assessed complete occlusion of a coronary artery for more than 3 months and with TIMI flow of 0. All patients had chronic total occlusions in one or more coronary arteries and subsequently underwent CTO PCI attempts upon clinical indication. We excluded patients with PCI attempts on bypass vessels only and misclassified patients with subtotal stenoses (90–99%) and a clear, narrow channel. In 196 patients, at least one femoral access was performed following puncture-site angiography. For these patients, we assessed femoral metrics and performed statistical analyses.

### 2.2. Assessment of Femoral Metrics

**Figure 1:**
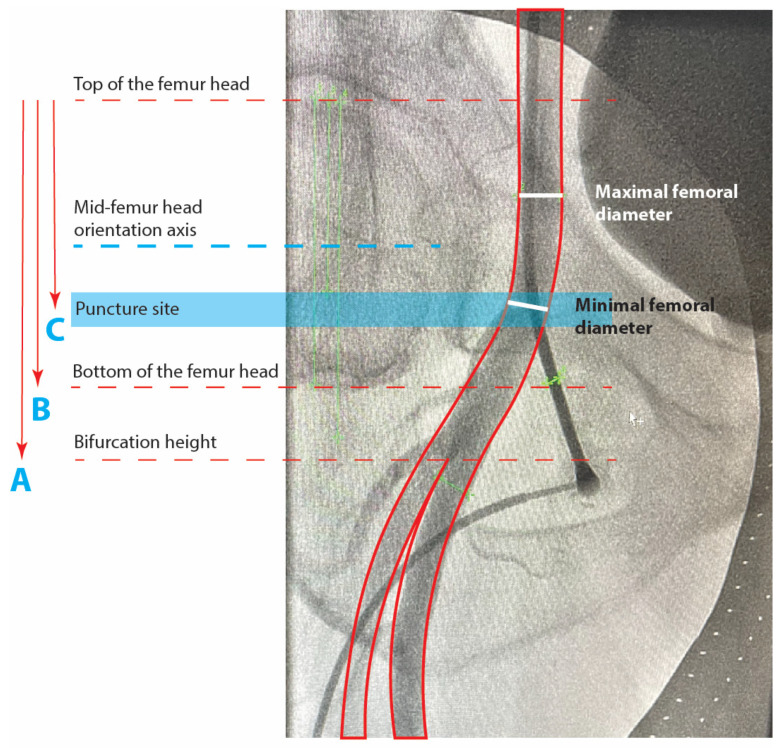
Puncture site angiography and femoral metrics.

Punctures of the femoral arteries were followed by angiographic control and documented accordingly. We performed measurements of the femoral angiography for each patient (Figure 1). The femoral artery was identified and its minimal and maximal diameter were measured. The maximal diameter was measured at a visually healthy artery position to indicate the reference vessel diameter. The minimal diameter was measured at the puncture site. Then, using the top of the femoral head as a reference point, we measured the length of the femoral head and the distances to the bifurcation and the puncture. The bifurcation and puncture site positions were then expressed as ratios to identify their position relative to the center of the femoral head. A value of 0.50 would mean a position of the bifurcation or puncture site at the center of the femoral head, and a value of more than 1 would indicate the bifurcation or puncture site to be lower than the femoral head position.

**Figure 2:**
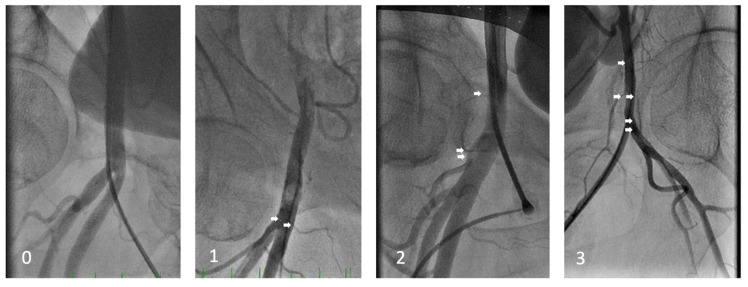
Side-branch density classification (0 to 3).

Minimal and maximal diameter, puncture site position, bifurcation, and side-branch density were assessed in 196 patients with available femoral angiography at the index procedure. All measurements were performed using Syngo Dynamics VA40 (© Siemens A.G., Munich, Germany) software, with the tenth-millimeter scale set automatically in a standardized manner for all patients. To verify the scale validity, we measured and verified the outer diameter of the catheter or the puncture set as a reference. Side branches to the femoral artery were also assessed using a score from 0 to 3 according to fluoroscopic visibility after contrast injection: 0 for no angiographic presence of side branches, 1 for up to two visible side branches, 2 for more than two side branches inside the area bordered by the top and bottom of the femoral head but with visually low vasculature density and 3 for a dense area of the vasculature of more than two side branches inside the area bordered by the top and bottom of the femoral head (Figure 2). This system aims to localize and quantify the presence of common femoral side branches at the puncture site, such as the superficial circumflex iliac artery, superficial epigastric artery, deep or superficial external pudendal artery, and the circumflex arteries. In patients with bifemoral access, the measurements were performed at the right femoral artery to maintain uniformity.

Minimal femoral diameter (millimeters) is measured at the visually most narrow position at the puncture site. Maximal femoral diameter (millimeters) is measured at a position where the femoral artery visually reaches the largest diameter, localized between the limits of the femoral head, at a position of a visually healthy artery with regular contours. Mid-femoral head-to-bifurcation is calculated in millimeters as C-(B/2), where C is the vertical distance between the top of the femoral head and the femoral bifurcation height and B is the vertical distance between the top and bottom limits of the femoral head. The bifurcation to femoral head height ratio is expressed as A/B, where A is the vertical distance between the bifurcation and the top of the femoral head. Values higher than 1 represent a bifurcation position distal to the lower limit of the femoral head. The puncture to femoral head height ratio is expressed as C/B and represents the puncture position relative to the vertical contours of the femoral head. Values higher than 0.5 represent a puncture more distal than the mid-femoral-head orientation axis.

This classification is determined by the number of visible side branches to the femoral artery between the top and bottom of the femoral head as the area of interest (white arrows), as well as visually assessed small vasculature density. Class 0 represents no visible side branches, 1 represents up to two visible side branches inside the area of interest, 2 more than two side branches without dense small vasculature, and 3 represents three or more side branches with visually increased density of small vasculature.

### 2.3. Data Collection and Procedural Endpoints

Data on baseline characteristics, medical history, laboratory reports and myocardial imaging reports were extracted from patient files at the cardiology department and the catheterization laboratory. The catheterization laboratory database also contained the procedural timelines, materials, procedural outcomes and other procedure-related information. Two study investigators obtained lesion characteristics and procedural data from angiographic images, and a consensus was reached on conflicting interpretations.

The primary endpoint included periprocedural and postprocedural complications during hospitalization. Secondary endpoints were the individual components of the primary endpoint. In-hospital complications were defined as the composite occurrence of major adverse cardiac and cerebrovascular events (MACCE—defined as all-cause in-hospital death, myocardial infarction (MI), ischemic stroke), coronary perforation, aortic dissection, pericardial tamponade, any acute surgery or bypass surgery during hospitalization, peripheral vascular complications and bleeding.

We defined coronary perforations as any contrast leak outside of the vessel contours persisting in more than one angiographic recording and requiring interventional treatment such as balloon inflation, coiling or covered stenting. Periprocedural bleeding was defined as a hemoglobin drop by 3 mg/dL after PCI, corresponding to the bleeding types 3 to 5 as defined by the Bleeding Academic Research Consortium [25]. Peripheral vascular complications were defined as the development of postprocedural hematoma with a diameter larger than 5 cm at the sheath insertion site requiring manual compression or serial clinical controls, pseudoaneurysm or the formation of an arteriovenous fistula after the procedure.

### 2.4. Statistical Analysis

Categorical variables were described by frequencies and percentages, and differences between groups were tested by Pearson’s Chi-2 test or Fisher’s exact test. Continuous variables were presented as mean ± standard deviation and compared using Student’s *t*-test or Mann–Whitney U test.

For our primary composite endpoint and secondary endpoints, group differences were estimated by Pearson’s Chi-2 test or Fisher’s exact test with a two-sided significance level alpha of 5%. Univariable logistic regression and multivariable adjustment were employed for the primary composite endpoint to identify predictors of the primary endpoint, initially excluding the femoral metrics. In the multivariable logistic regression, the significant variables were additionally force-adjusted for age. An additional multivariable logistic regression model included femoral access site metrics and adjusted for body surface area (BSA). Finally, we performed a 1:1 nearest-neighbor propensity score matching to neutralize the group differences, including matching variables that exhibited a statistical significance level of *p* < 0.1 with a caliper of 0.2. All baseline and procedural variables were included and derived groups were compared for in-hospital outcomes. All statistical analyses were performed with SPSS 25.0 for Mac (SPSS Inc, IBM, New York, NY, USA) and R Studio (Version 2024.09.0+375, Posit Software) using the Bioconductor and CRAN packages.

## 3. Results

### 3.1. Baseline Characteristics

The baseline characteristics are shown in Table 1. Of 271 procedures, 18.1% were female; the mean age was 67.6 ± 11.2 years, with significantly older women than men (71.59 ± 12.02 vs. 66.68 ± 10.86, *p* = 0.005). Women had fewer prior coronary interventions than men (44.9% vs. 65.8%, *p* = 0.006), although they had similar prior myocardial infarction rates (42.9% vs. 48.6%, *p* = 0.463). Otherwise, the two study groups had comparable baseline clinical characteristics.

### 3.2. Lesion and Procedural Characteristics

Lesion and procedural characteristics are presented in Table 2. The lesion characteristics and complexity scores in women and men were not significantly different, with similar J-CTO and PROGRESS-CTO scores. Retrograde attempts were performed equally frequently in women and men (18.4% vs. 23%, *p* = 0.482). Women had a more frequent use of rotational atherectomy than men (12.2% vs. 4.5%, *p* = 0.048). Success rates were not significantly different between women and men (75.5% vs. 82%; *p* = 0.298) and neither were procedural times (180 ± 65 vs. 187 ± 67 min, *p* = 0.511), fluoroscopy duration (55 ± 34 vs. 54 ± 35 min, *p* = 0.975) and contrast volumes (232 ± 101 vs. 262 ± 116 mL, *p* = 0.092). However, the total dose area product was significantly lower in women than in men (14,590 vs. 19,314 cGy cm^2^, *p* = 0.024).

### 3.3. Femoral Artery Metrics

The transfemoral approach was the preferred main access route in 196 (72.3%) patients, of which 35 were female (71.4%). Women had a significantly smaller minimal femoral diameter at the femoral puncture site (5.9 ± 1.2 mm vs. 7.6 ± 1.4 mm, *p* < 0.001) and smaller maximal reference femoral diameter (6.5 ± 1.2 mm vs. 8.3 ± 1.6 mm, *p* < 0.001). The femoral artery bifurcation had a lower distance to the center of the femoral head in women as compared to men (19.5 ± 12.5 mm vs. 24.3 ± 12.5 mm, *p* = 0.048). It was typically located inside the height of the femoral head, as expressed by the ratio of the bifurcation height to femoral head length (0.95 ± 0.30 mm vs. 1.02 ± 0.27 mm, *p* = 0.197).

### 3.4. Procedural and In-Hospital Complications

The occurrence of in-hospital complications and the individual components of the primary endpoint are presented in Table 3. The overall study cohort had an in-hospital complication rate of 8.5%, with significantly more complications in women as compared to men (16.3% vs. 6.8%; *p* = 0.044). The in-hospital MACCE rate was 2.6% without significant differences between groups (6.1% vs. 1.8%; *p* = 0.114). Among the individual components, major bleeding was the most common (3.0%), followed by peripheral vascular complications (2.2%) and coronary perforations (2.2%). Four intervention attempts (1.5%) were followed by acute surgery due to complications—twice a surgical treatment of pericardial tamponade, an aortic surgery due to dissection of the ascending aorta and an acute surgery due to retroperitoneal bleeding. The higher complications in women were numerically driven by major bleeding (6.1% vs. 2.3%; *p* = 0.160) and peripheral vascular complications (4.1% vs. 1.8%, *p* = 0.297).

### 3.5. Predictors for In-Hospital Complications

All parameters were entered into an univariable logistic regression model, and the results are presented in Supplementary Table S1. Sex was correlated to the primary endpoint, with women showing a lower in-hospital complication risk than men (OR = 2.70; CI 1.02 to 6.71; *p* = 0.035). Furthermore, the J-CTO score (OR 1.46; 95% CI 1.04 to 2.04; *p* = 0.027) and proximal cap ambiguity (OR = 2.78, CI 1.16 to 6.68; *p* = 0.022) showed a positive correlation with in-hospital complications.

Patient age and parameters significantly correlating with procedural complications were entered into a multivariable logistic regression model (Supplementary Table S2). Sex alone preserved the significant correlation with the composite primary endpoint in favor of female patients (OR = 2.92; CI 1.07 to 7.60; *p* = 0.024). After adjusting for femoral artery metrics and body surface area (BSA), sex lost the correlation significance to the primary endpoint, with maximal reference femoral diameter (OR = 0.30; CI 0.17 to 0.50; *p* < 0.001) and femoral side branch density (OR = 2.45, CI 1.26 to 5.20; *p* = 0.012) showing adjusted significant correlations to in-hospital complications.

### 3.6. Propensity Score-Matched Analysis

In a 1:1 propensity score-matched cohort of 45 female and 45 male patients, baseline clinical and angiographic characteristics were balanced using baseline and procedural variables with a *p*-Value < 0.1 (Supplementary Table S4). Female patients continued to experience a higher rate of in-hospital complications compared to male patients (17.8% vs. 0.0%, *p* = 0.006), with vascular complications and major bleeding driving the results. Differences in femoral metrics persisted with women showing significantly smaller minimal femoral artery diameters compared to male patients (5.99 ± 1.12 vs. 7.54 ± 1.23 mm, *p* < 0.001) as well as smaller maximal reference femoral diameters (6.51 ± 1.18 vs. 8.15 ± 1.33 mm, *p* < 0.001). The mid-femoral head to bifurcation distance was shorter in female patients compared to male patients (18.9 [9.03; 32.4] vs. 27.4 [21.2; 37.9] mm, *p* = 0.013).

## 4. Discussion

The main findings of this study are as follows: (1) Female sex is associated with worse in-hospital outcomes after contemporary CTO PCI. (2) Higher rates of major bleeding and peripheral vascular complications primarily contributed to the elevated in-hospital complication rates in women. (3) Female patients had smaller femoral vessels and a higher femoral bifurcation. (4) Femoral artery reference diameter and femoral side-branch density were independent predictors of in-hospital complications.

In general, women undergoing PCI may have an increased risk for in-hospital complications, but with evolving PCI procedures, recent data indicate diminishing sex disparities in outcomes among all-comer PCI cohorts [26,27,28]. In the context of CTO PCI, prospective gender studies are scarce, with existing large observational studies yielding ambiguous results [10,14,19,29]. A meta-analysis found no difference in complication rates after CTO PCI in men and women. However, the composite endpoint did not include complications such as coronary perforations and vascular complications [18]. In our contemporary CTO PCI cohort, women experienced significantly higher rates of in-hospital complications than men, in line with more recent studies [30,31,32,33]. This suggests the ongoing relevance of sex-related disparities in procedural outcomes and highlights the need for further exploration into the underlying mechanisms of this.

Higher complication rates after complex coronary procedures in women have been typically linked to differences in clinical baseline characteristics [16]. However, in our cohort, apart from older age, baseline characteristics were comparable between women and men, indicating that baseline clinical status alone likely does not account for the observed differences. The increased complication rates in women were mainly driven by major bleeding and peripheral vascular complications. Indeed, women undergoing CTO PCI are known to have increased bleeding risk [34,35], primarily due to access-site complications from catheter manipulation. Of note, our cohort had a high proportion of patients already loaded with ticagrelor or prasugrel due to prior acute coronary events, but without differences between sexes. For this reason, it is unlikely that the use of stronger antithrombotic agents other than the standard clopidogrel influenced the results. However, the older age in female patients may have influenced higher rates of major bleeding, as an older age is described to be associated with higher bleeding risk after PCI [36]. Nevertheless, sex-specific factors remain influential independently of age [36]. Importantly, our propensity score-matched cohort suggested that sex-related differences in outcomes and their statistical significance persisted even after rigorous balancing of the baseline and procedural characteristics.

We believe that commonly unconsidered parameters such as coronary artery size, vascular access characteristics, and existing revascularization strategies based dominantly on male patient populations may have affected outcomes in female patients. To test this hypothesis, we conducted detailed assessments of femoral artery access anatomy in our patient cohort undergoing CTO PCI. Radial access is generally the primary recommended access route for coronary interventions, as femoral access is associated with worse outcomes [37]. However, CTO PCI often necessitates transfemoral access, as two access routes are needed to facilitate dual injections and retrograde wiring. The radial-femoral or bifemoral approach provides ergonomic advantages for the operators during lengthy procedures and enables the use of a broader range of devices requiring larger guiding catheters.

The hypothesis that the femoral artery characteristics and the puncture site location can predict in-hospital complications after CTO PCI has not previously been explored. In our cohort, we systematically measured femoral artery anatomy and identified significant associations between femoral artery metrics and in-hospital complications, including bleeding and peripheral vascular complications. The reference diameter of the femoral artery was smaller in women than in men and independently predicted complications after counting for potential confounders like sheath size and antithrombotic agents. This observation can be explained in two possible ways: either femoral artery anatomy directly influences access-site complications, or it acts as a surrogate marker reflecting the dimensions of the coronary artery tree. The latter is vaguely described in the literature, but it has been previously described that the femoral artery and coronary sizes are correlated to similar baseline characteristics, such as sex and body size [20,38,39,40]. Consequently, caution is advisable when managing patients with smaller femoral arteries, not only during puncture but also during coronary artery manipulation. Of note, these interpretations are hypothetical and, due to the study design, not yet conclusive.

As for the characteristics of the transfemoral access route, the finding of smaller femoral artery diameters in women aligns with previously published data [20]. In addition, we observed that femoral bifurcations were positioned higher in women compared to men, potentially increasing the risk of puncturing below the bifurcation—a scenario associated with greater complications and less effective compression [41]. This insight underscores the importance of careful consideration of the hypothetical femoral bifurcation positioning, particularly in female patients, when attempting femoral access [22]. Rather than relying solely on visual estimation and fluoroscopic landmarks, pre-procedural imaging of femoral artery anatomy could be advantageous for women undergoing CTO PCI. Assessing vessel diameter, bifurcation height, and the density of side branches at the puncture site might facilitate access planning and help reduce complications. Using ultrasound to assess these parameters at the femoral access site before ultrasound-guided puncture can shape access strategies. For example, due to smaller femoral artery diameters or higher bifurcations, one may change the puncture site (left or right femoral artery), reconsider puncture height or opt to use sheath-less guiding catheters. We emphasize the benefits of standardized and structured approaches, particularly in less-experienced centers, as it can contribute to the learning curve and minimize procedural risks. To ensure external reproducibility, our findings require validation across multiple centers, given the considerable variability in CTO PCI outcomes influenced by procedural volume, operator experience, availability of dedicated devices and individual access strategy preferences. While our study was conducted at a single center with percutaneous access performed predominantly by experienced CTO operators, this allowed for consistency in access approaches and emphasized the importance of femoral artery anatomy, especially in women. The high proportion of femoral access indicates a more liberal use by expert operators in this context. Furthermore, female sex did not influence the access choice or adaption of the puncture technique, but future consideration of sex-specific anatomical differences remains important. As for intravascular imaging, its use was limited to imaging-guided antegrade puncture and avoiding side-branch closure during dissection and re-entry in CTOs including bifurcations. Nevertheless, we encourage intravascular imaging-directed vessel characterization and stent optimization, which could probably reduce coronary complications and improve results.

After adjusting for femoral artery metrics, female sex no longer independently predicted in-hospital complications. Hence, the previously noted association between female sex and in-hospital complications may be mediated by anatomical differences at the femoral access site. These findings highlight the importance of carefully considering femoral artery characteristics—particularly vessel size, bifurcation height and side-branch density—when planning access strategies for female patients undergoing CTO PCI.

But beyond these anatomical factors, analyses at the biomolecular level are still lacking and the influence of hormonal status or other physiological mechanisms on procedural outcomes remains unexplored. Given the findings of this study, we speculate that, despite advances in interventional strategies, techniques and devices—even with similar baseline profiles—women demonstrate more vulnerability to in-hospital complications as compared to men. Therefore, adapting percutaneous access protocols to account for female-specific anatomical considerations could potentially mitigate procedural risks. Further dedicated studies in this context could provide insights into the mechanisms behind sex disparities in patients undergoing complex coronary interventions such as CTO PCI.

## 5. Limitations

Our study is limited by its observational, single-center design. First, statistical analysis is constrained by its post hoc nature and the small number of participants compared to larger observational studies. Nevertheless, we were able to extract and analyze a detailed set of clinical, angiographic and procedural data from our patients. We also aimed to identify procedural information from angiographic images that were not routinely documented in patient files. The study endpoint encompasses non-access-site- and access-site-related complications. Although the latter were dominant and drove the results, this is a major limitation, as the composite endpoint was considered to derive interpretations related to femoral access site characteristics. Second, we could not report the percentage of ultrasound-guided femoral puncture. Third, femoral artery assessments were performed using two-dimensional angiographic images, potentially introducing measurement inaccuracies due to projection. Ideally, computed tomography would serve as the standard imaging modality to achieve precise vessel dimension measurements; however, such imaging is not part of routine practice.

Additionally, despite a careful definition, the visual estimation of femoral side-branch density could be affected by variations in image quality and the visibility of small vessels. Although our standardized imaging approach might introduce potential biases, we believe the quantification of side-branch density remains clinically valuable. Finally, our analysis documented only the occurrence of bleeding and peripheral vascular complications without further stratification using scoring systems. Future research incorporating a more granular classification of complications could provide additional insights into procedural risk factors and outcomes.

## 6. Conclusions

Our findings suggest that female sex is associated with in-hospital complications after CTO PCI. Smaller reference vessel diameter and increased side-branch density at the femoral access site emerged as potential contributors to procedural risk. These findings suggest that anatomical differences in femoral access may partially explain sex disparities in procedural outcomes. Assessing these variables may aid in risk stratification and access planning. Larger prospective and multicenter studies are required to confirm our findings and inform standardized access strategies.

## 7. Clinical Implication

Our findings underscore the value of pre-procedural imaging of the femoral artery to optimize access in patients undergoing CTO PCI, particularly women. Reliance on fluoroscopic landmarks alone may be insufficient in cases of high bifurcation, dense side-branching, or small-caliber vessels—features more frequently observed in female patients. Incorporating routine ultrasound guidance and structured access protocols into procedural planning could enhance puncture precision and reduce complications.

## Figures and Tables

**Table 1:** Baseline clinical characteristics.

Variable	Total Cohort (*n* = 271)	Male (*n* = 222)	Female (*n* = 49)	*p*-Value
Age	67.56 ± 11.22	66.68 ± 10.86	71.59 ± 12.02	0.005
BMI	28.8 ± 6.10	28.5 ± 6.09	30.2 ± 5.98	0.075
Obesity	94 (34.7)	79 (35.6)	15 (30.6)	0.508
Diabetes	99 (36.5)	83 (37.4)	16 (32.7)	0.533
Hypertension	197 (72.7)	160 (72.1)	37 (75.5)	0.625
Current smoker	78 (28.8)	68 (30.6)	10 (20.4)	0.153
Family history of premature CAD	71 (26.1)	58 (26.1)	13 (26.5)	0.954
Prior MI	129 (47.6)	108 (48.6)	21 (42.9)	0.463
Prior PCI	168 (62)	146 (65.8)	22 (44.9)	0.006
Heart Failure	45 (16.6)	35 (15.8)	10 (20.4)	0.429
Prior CABG	37 (13.7)	30 (13.5)	7 (14.3)	0.887
PAD	51 (18.8)	40 (18.0)	11 (22.4)	0.606
Stroke/TIA	17 (6.27)	13 (5.86)	4 (8.16)	0.521
Chronic pulmonary disease	33 (12.2)	24 (10.8)	9 (18.4)	0.143
CKD	59 (21.8)	49 (22.1)	10 (20.4)	0.798
Anticoagulation therapy	53 (19.6)	45 (20.3)	8 (16.3)	0.666
Prasugrel/Ticagrelor	59 (21.8)	47 (21.2)	12 (24.5)	0.750
GFR (mL/min/1.73 m^2^)	69.8 ± 24.6	70.5 ± 24.4	66.2 ± 25.1	0.303
Leucocytes (G/L)	7.53 [6.52; 9.80]	7.50 [6.44; 9.86]	7.91 [6.72; 9.34]	0.687
Hemoglobin (g/dL)	13.2 ± 1.97	13.2 ± 1.97	12.9 ± 1.98	0.279
LDL (mg/dL)	59.9 [46.0; 78.6]	58.3 [46.1; 78.3]	65.4 [45.9; 78.6]	0.793
NT-proBNP (ng/dL)	501 [203; 1518]	536 [226; 1514]	478 [146; 1746]	0.317
CRP (mg/L)	0.23 [0.08; 0.63]	0.23 [0.08; 0.56]	0.23 [0.11; 0.76]	0.359

CAD—coronary artery disease; MI—myocardial infarction; PCI—percutaneous coronary intervention; CABG—coronary artery bypass grafting; PAD—peripheral artery disease; TIA—transient ischemic attack; CKD—chronic kidney disease; GFR—glomerular filtration rate (MDRD-GFR); LDL—low density lipoprotein; NT-proBNP—N-terminal pro-B-type natriuretic peptide; CRP—C-reactive protein.

**Table 2:** Angiographic and procedural characteristics.

Variable	Total Cohort (*n* = 271)	Male (*n* = 222)	Female (*n* = 49)	*p*-Value
Number of diseased vessels				
1	38 (14)	29 (13.1)	9 (18.4)	0.333
2	98 (36.2)	79 (35.6)	19 (38.8)	0.674
3	135 (49.8)	114 (51.4)	21 (42.9)	0.282
CTO localization				
LAD	67 (24.7)	53 (23.9)	14 (28.6)	0.501
RCX	51 (18.8)	43 (19.4)	8 (16.3)	0.613
RCA	152 (56.1)	125 (56.3)	27 (55.1)	0.852
J-CTO	1.75 ± 1.240	1.76 ± 1.219	1.69 ± 1.342	0.731
PROGRESS-CTO	0.77 ± 0.804	0.78 ± 0.796	0.71 ± 0.842	0.609
In-stent CTO	22 (8.1)	19 (8.6)	3 (6.1)	0.572
Bridging collateral	135 (49.8)	113 (50.9)	22 (44.9)	0.447
Blunt stump	124 (45.8)	104 (46.8)	20 (40.8)	0.443
Calcification	66 (24.4)	51 (23)	15 (30.6)	0.259
Length > 20 mm	170 (62.7)	145 (65.3)	25 (51.0)	0.061
Proximal cap ambiguity	103 (38.0)	87 (39.2)	16 (32.7)	0.394
Retry	33 (12.2)	26 (11.7)	7 (14.3)	0.618
Tortuosity	26 (9.6)	21 (9.5)	5 (10.2)	0.873
CTO involving a bifurcation lesion	55 (20.3)	50 (22.5)	5 (10.2)	0.052
Largest sheath size (F)				0.430
6	119 (43.9)	93 (41.9)	26 (53.1)	
7	141 (52.0)	120 (54.1)	21 (42.9)	
8	10 (3.69)	8 (3.60)	2 (4.08)	
14	1 (0.37)	1 (0.45)	0 (0.0)	
Retrograde	60 (22.1)	51 (23.0)	9 (18.4)	0.482
Rotational atherectomy	16 (5.9)	10 (4.50)	6 (12.2)	0.048
Intravascular imaging:				0.689
IVUS	27 (10.0)	24 (10.8)	3 (6.1)	
OCT	3 (1.1)	3 (1.4)	0 (0.0)	
Femoral	196 (72.3)	161 (72.5)	35 (71.4)	0.999
Technical success	219(80.8)	182(82.0)	37(75.5)	0.298
Contrast volume (ml)	257.07 ± 114.07	262.75 ± 116.21	231.98 ± 101.42	0.092
Total DAP (cGycm^2^)	18,427 ± 12,840	19,314 ± 13,255	14,590 ± 10,119	0.024
Fluoroscopy time (min)	54.60 ± 34.78	54.56 ± 35.12	54.74 ± 33.63	0.975
Total procedural time (min)	185.75 ± 66.77	187.02 ± 67.15	180.06 ± 65.42	0.511
**Femoral metrics**	***n* = 196**	***n* = 161**	***n* = 35**	
Minimal femoral diameter (mm)	7.3 ± 1.5	7.6 ± 1.4	5.9 ± 1.2	<0.001
Maximal femoral diameter (mm)	8.0 ± 1.7	8.3 ± 1.6	6.5 ± 1.2	<0.001
Femoral side branches density	1.2 ± 0.9	1.2 ± 0.9	1.5 ± 0.8	0.054
Mid-femoral head to bifurcation (mm)	23.5 ± 12.6	24.3 ± 12.5	19.5 ± 12.5	0.048
Bifurcation to femoral head height ratio	1.01 ± 0.28	1.02 ± 0.27	0.95 ± 0.30	0.197
Puncture to femoral head height ratio	0.58 ± 0.28	0.59 ± 0.26	0.54 ± 0.36	0.501

CTO—chronic total occlusion; LAD—left anterior descending; RCX—ramus circumflexus; RCA—right coronary artery; J-CTO—Japan-CTO; IVUS—intravascular ultrasound; OCT—optical coherence tomography; DAP—dose-area product.

**Table 3:** Procedural complications.

Variable	Total Cohort (*n* = 271)	Male (*n* = 222)	Female (*n* = 49)	*p*-Value
In-hospital complications	23 (8.5)	15 (6.8)	8 (16.3)	0.044
MACCE	7 (2.6)	4 (1.8)	3 (6.1)	0.114
Aorta dissection	3 (1.1)	3 (1.4)	0 (0.0)	1.00
Coronary perforation with need for intervention	6 (2.2)	5 (2.3)	1 (2.0)	1.00
Tamponade	1 (0.4)	0	1 (2.0)	0.181
Peripheral vascular complication	6 (2.2)	4 (1.8)	2 (4.1)	0.297
Major bleeding	8 (3.0)	5 (2.3)	3 (6.1)	0.160
Stroke	3 (1.1)	2 (0.9)	1 (2.0)	0.452
In-hospital death	4 (1.5)	2 (0.9)	2 (4.1)	0.151
Acute surgery	4 (1.5)	3 (1.4)	1 (2.0)	0.717
Aortic dissection surgery	1	1	0	
Pericardial tamponade surgery	2	1	1	
Retroperitoneal bleeding	1	1	0	

MACCE—major adverse cardiovascular and cerebrovascular events.

## Data Availability

Data could be shared by the principal investigator upon reasonable request.

## Supplementary Materials

The following supporting information can be downloaded at: https://www.mdpi.com/article/10.3390/jcm14134496/s1, Figure S1: Flowchart of the study, Table S1: Univariable logistic regression, Table S2: Multivariable logistic regression, Table S3: Multivariable logistic regression including femoral access site metrics, Table S4: Propensity score 1:1 matched cohort.
