# Supplementary material for: Role of Femoral Artery Access Characteristics and Female Sex in In-Hospital Complications for Patients Undergoing Recanalization of Chronic Total Occlusions
Source: J Clin Med. 2025 Jun 25;14(13):4496. doi: 10.3390/jcm14134496 (PMC12250262; doi:10.3390/jcm14134496)
Supplement: Supplementary file 1 [file jcm-14-04496-s001.zip › jcm-3587783-supplementary.pdf]

SUPPLEMENTARY MATERIAL

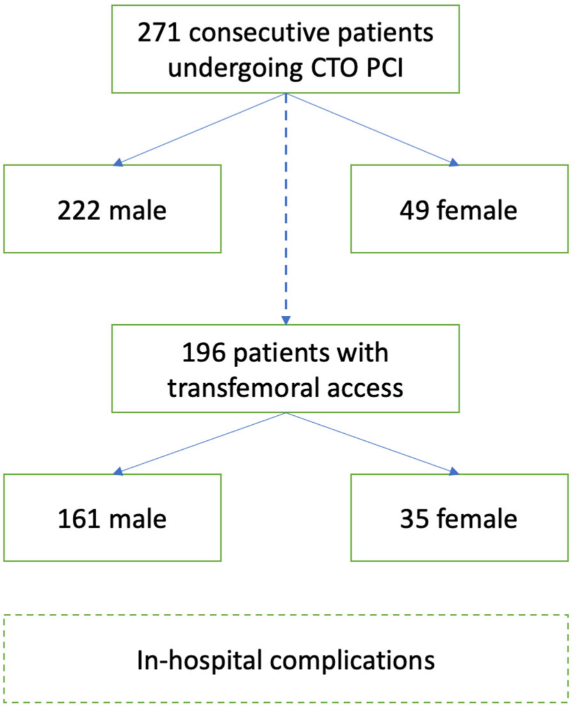

Figure S1: Flowchart of the study

Table S1: Univariable logistic regression

Variable	OR	CI (95%)		P Value
Female sex	**2.70**	**1.02**	**6.71**	**.035**
Age	1.01	0.97	1.05	.631
BMI	1.01	0.95	1.08	.700
Obesity	1.01	0.41	2.46	.992
Diabetes	0.74	0.29	1.87	.527
Hypertension	0.85	0.33	2.15	.725
Current smoker	0.86	0.33	2.28	.766
Family history of CAD	0.77	0.27	2.15	.612
Prior MI	0.83	0.35	1.97	.679
Prior PCI	0.95	0.40	2.28	.908
Prior CABG	1.37	0.44	4.28	.587
Heart Failure	0.45	0.10	2.01	.298
COPD	2.18	0.75	6.34	.151
CKD	0.74	0.24	2.26	.596
Number of diseased vessels	0.98	0.54	1.78	.943
CTO vessels	1.03	0.44	2.40	.950
In-stent CTO	0.49	0.06	3.83	.498
CTO involving a bifurcation lesion	1.82	0.71	4.68	.212
Bridging collateral	1.63	0.68	3.91	.271
Blunt stump	1.33	0.56	3.12	.519
Calcification	1.75	0.71	4.33	.228
Length > 20 mm	1.13	0.46	2.76	.797
Retry	1.09	0.31	3.89	.894
J-CTO score	1.46	1.04	2.04	**.027**
Proximal cap ambiguity	2.78	1.16	6.68	**.022**

**Moderate-severe tortuosity**	2.16	0.68	6.92	.194
**PROGRESS-CTO score**	1.44	0.88	2.36	.150
**Good distal landing zone**	0.63	0.27	1.50	.297
**Number of stents**	1.08	0.78	1.50	.634
**Retrograde**	2.01	0.81	5.00	.133
**Rotational atherectomy**				
**Contrast volume (ml)**	1.00	1.00	1.00	.777
**Total DAP (cGycm²)**	1.00	1.00	1.00	.452
**Fluoroscopy duration (min)**	1.01	1.00	1.02	.293
**Procedural time (min)**	1.00	1.00	1.01	.164
**Technical success**	0.51	0.20	1.30	.159
**Femoral**	1.38	0.52	4.41	.530
**BMI**	1.03	0.96	1.09	.416
**PAD**	2.04	0.74	5.15	.161
**Stroke/TIA**	0.74	0.03	3.96	.777
**CKD**	0.76	0.21	2.15	.628
**Anticoagulation therapy**	0.88	0.24	2.49	.824
**Prasugrel/Ticagrelor**	1.66	0.60	4.16	.308
**GFR (mL/min/1.73 m²)**	1.01	0.99	1.03	.264
**Leucocytes (G/L)**	0.95	0.84	1.09	.489
**Hemoglobin (g/dL)**	0.91	0.73	1.13	.390
**LDL (mg/dL)**	1.00	0.98	1.01	.565
**NT-proBNP (ng/dL)**	1.00	1.00	1.00	.585
**CRP (mg/L)**	0.90	0.61	1.34	.618
**Rotational atherectomy**	–	–	–	–
**Largest sheath (F)**	1.21	0.76	1.94	.427
**Intravascular imaging**	-	-	-	-
**Femoral metrics**				
**Minimal femoral diameter (mm)**	0.98	0.71	1.38	.915
**Maximal femoral diameter (mm)**	0.34	0.22	0.53	**<.001**
**Femoral side branches density**	2.30	1.32	4.02	**.003**
**Mid-femoral head to bifurcation (mm)**	0.97	0.93	1.00	.083
**Bifurcation to femoral head height ratio**	0.23	0.04	1.38	.109
**Puncture to femoral head height ratio**	2.16	0.39	12.10	.380

BMI – body mass index; CAD – coronary artery disease; MI – myocardial infarction; PCI – percutaneous coronary intervention; CABG – coronary artery bypass grafting; COPD – chronic pulmonary disease; CKD – chronic kidney disease; DAP – dose-area product; PAD – peripheral artery disease; TIA – transient ischemic attack; CKD – chronic kidney disease; GFR – glomerular filtration rate (MDRD-GFR); LDL – low density lipoprotein; NT-proBNP – N-terminal pro-B-type natriuretic peptide; CRP – C-reactive protein

Table S2: Multivariable logistic regression

Variable	OR	CI (95%)		P Value
**Female sex**	2.92	1.065	7.602	**.024**
**Age**	1.005	.967	1.047	.790
**J-CTO score**	1.28	.880	1.866	.193
**Proximal cap ambiguity**	2.29	.863	6.343	.101

Table S3. Multivariable logistic regression including femoral access site metrics

Variable	P Value	OR	CI (95%)	
**Female sex**	.732	1.321	.252	6.448
**Age**	.207	1.039	.981	1.047

**J-CTO score**	.270	1.389	.772	1.866
**Proximal cap ambiguity**	.052	4.270	1.042	20.433
**Maximal femoral diameter (mm)**	**<.001**	0.303	.165	.502
**Femoral side branches density**	**.012**	2.452	1.258	5.203
**Body surface area (BSA)**	.387	4.734	0.121	0.154

Table S4. Propensity score 1:1 matched cohort. Matching variables: Age, BMI, prior PCI, Length > 20mm, CTO involving a bifurcation lesion, Rotational atherectomy, total DAP, Contrast volume (p-Values < 0.1)

Variable	All (N=90)	Male (N=45)	Female (N=45)	P Value
**BASELINE**				
**Age**	74.5±11.3	74.7±10.9	74.3±11.9	.883
**BMI**	27.0 [24.3;30.5]	26.6 [23.8;29.7]	27.3 [24.8;31.2]	.401
**Obesity**	24(26.7)	10(22.2)	14(31.1)	.475
**Diabetes**	32(35.6)	19(42.2)	13(28.9)	.271
**Hypertension**	70(77.8)	36(80.0)	34(75.6)	.8
**Current smoker**	18(20.0)	8(17.8)	10(22.2)	.792
**Family history of premature CAD**	20(22.2)	8(17.8)	12(26.7)	.447
**Prior MI**	38(42.2)	19(42.2)	19(42.2)	1.0
**Prior PCI**	47(52.2)	26(57.8)	21(46.7)	.399
**Heart Failure**	17(18.9)	7(15.6)	10(22.2)	.59
**Prior CABG**	16(17.8)	10(22.2)	6(13.3)	.408
**PAD**	20(22.2)	10(22.2)	10(22.2)	1.0
**Stroke/TIA**	6(6.67)	4(8.89)	2(4.44)	.677
**Chronic Pulmonary Disease**	14(15.6)	6(13.3)	8(17.8)	.771
**CKD**	21(23.3)	11(24.4)	10(22.2)	1.0
**Anticoagulation therapy**	18(20.0)	10(22.2)	8(17.8)	.792
**GFR (mL/min/1.73 m2)**	71.4±24.3	76.0±22.9	67.0±25.0	.098
**Leucocytes (G/L)**	7.38 [6.60;9.57]	7.05 [6.52;9.46]	8.01 [6.76;9.80]	.238
**Hemoglobin (g/dL)**	13.1±1.94	13.3±1.87	12.8±2.01	.256
**LDL (mg/dL)**	57.6 [45.3;75.1]	53.2 [47.0;72.0]	65.4 [44.9;82.4]	.666
**NT-proBNP (ng/dL)**	331 [145;1059]	316 [148;749]	490 [147;1615]	.309
**CRP (mg/L)**	0.19 [0.09;0.82]	0.17 [0.08;0.66]	0.23 [0.11;0.86]	.359
**Minimal femoral diameter (mm)**	6.79±1.41	7.54±1.23	5.99±1.12	<.001
**Maximal femoral diameter (mm)**	7.35±1.50	8.15±1.33	6.51±1.18	<.001
**Femoral side branches density**	1.29 (0.91) 0.193	1.15±0.99	1.44±0.80	.193
**Mid-femoral head to bifurcation (mm)**	24.6 [14.3;35.4]	27.4 [21.2;37.9]	18.9 [9.03;32.4]	.013
**Puncture to femoral head height ratio**	0.57±0.29	0.62±0.23	0.52±0.34	.199
**PROCEDURAL**				
**Number of diseased vessels**				.105
**1**	14 (15.6)	5 (11.1)	9 (20.0)	
**2**	30 (33.3)	12 (26.7)	18 (40.0)	
**3**	46 (51.1)	28 (62.2)	18 (40.0)	
**CTO vessel:**				.967
**LAD**	21 (23.3)	10 (22.2)	11 (24.4)	
**CX**	16 (17.8)	8 (17.8)	8 (17.8)	
**RCA**	53 (58.9)	27 (60.0)	26 (57.8)	
**In-stent CTO**	4(4.44)	2(4.44)	2(4.44)	1.0
**Bridging collateral**	51(56.7)	29(64.4)	22(48.9)	.202
**Blunt stump**	32(35.6)	13(28.9)	19(42.2)	.271
**Calcification**	8(8.89)	0(0.00)	8(17.8)	.006
**Length >20 mm**	48(53.3)	25(55.6)	23(51.1)	.833
**Proximal cap ambiguity**	28(31.1)	12(26.7)	16(35.6)	.495
**Largest sheath (F)**				0.394
**6**	43 (47.8%)	18 (40.0%)	25 (55.6%)	
**7**	44 (48.9%)	25 (55.6%)	19 (42.2%)	
**8**	3 (3.33%)	2 (4.44%)	1 (2.22%)	
**Retry**	9(10.0)	3(6.67)	6(13.3)	.485

**CTO involving a bifurcation Lesion**	12(13.3)	7(15.6)	5(11.1)	.756
**Retrograde**	16(17.8)	7(15.6)	9(20.0)	.783
**Femoral**	66(73.3)	34(75.6)	32(71.1)	.812
**Technical success**	69(76.7)	36(80.0)	33(73.3)	.618
**Fluoroscopy time (min)**	169 [135;199]	161 [135;185]	170 [135;201]	.366
**J-CTO**	1.00 [0.25;2.00]	1.00 [0.00;2.00]	2.00 [1.00;3.00]	.215
**PROGRESS-CTO**	0.00 [0.00;1.00]	0.00 [0.00;1.00]	1.00 [0.00;1.00]	.261
**Contrast volume (ml)**	232±104	234±104	230±104	.865
**Total DAP**	12802 [7866;19986]	13702 [8533;20300]	11800 [7838;19687]	.72
**Total procedural time (min)**	169 [135;199]	161 [135;185]	170 [135;201]	.366
**OUTCOMES**				
**In-hospital complications**	8(8.89)	0(0.00)	8(17.8)	.006
**MACCE**	3(3.33)	0(0.00)	3(6.67)	.242
**Aorta dissection**	0(0.00)	0(0.00)	0(0.00)	.
**Coronary perforation with need for intervention**	1(1.11)	0(0.00)	1(2.22)	1.0
**Tamponade**	1(1.11)	0(0.00)	1(2.22)	1.0
**Peripheral vascular complication**	2(2.22)	0(0.00)	2(4.44)	.494
**Major bleeding**	3(3.33)	0(0.00)	3(6.67)	.242
**Stroke**	1(1.11)	0(0.00)	1(2.22)	1.0
**In-hospital death**	2(2.22)	0(0.00)	2(4.44)	.494
**Acute surgery**	1(1.11)	0(0.00)	1(2.22)	1.0
**PSM distance**	0.26 [0.15;0.40]	0.26 [0.15;0.39]	0.25 [0.15;0.40]	0.806

CAD – coronary artery disease; MI – myocardial infarction; PCI – percutaneous coronary intervention; CABG – coronary artery bypass grafting; PAD – peripheral artery disease; TIA – transient ischemic attack; CKD – chronic kidney disease; GFR – glomerular filtration rate (MDRD-GFR); LDL – low-density lipoprotein; NT-proBNP – N-terminal pro-B-type natriuretic peptide; CRP – C-reactive protein; CTO – chronic total occlusion; LAD – left anterior descending artery; RCX – ramus circumflexus; RCA – right coronary artery; J-CTO – Japan-CTO score; IVUS – intravascular ultrasound; OCT – optical coherence tomography; DAP – dose-area product; MACCE – major adverse cardiovascular and cerebrovascular events.
